# Four Types of RNA Modification Writer-Related lncRNAs Are Effective Predictors of Prognosis and Immunotherapy Response in Serous Ovarian Carcinoma

**DOI:** 10.3389/fimmu.2022.863484

**Published:** 2022-05-02

**Authors:** Lele Ye, Kan Pan, Su Fang, Su-Ni Wu, Su Chen, Sangsang Tang, Nan Wang, Haoke Zhang, Xinya Tong, Xinyu Shi, Shiyu Feng, Dan Xiang, Ruanmin Zou, Yingying Hu, Xiangyang Xue, Gangqiang Guo

**Affiliations:** ^1^ Wenzhou Collaborative Innovation Center of Gastrointestinal Cancer in Basic Research and Precision Medicine, Wenzhou Key Laboratory of Cancer-related Pathogens and Immunity, Department of Microbiology and Immunology, Institute of Molecular Virology and Immunology, Institute of Tropical Medicine, School of Basic Medical Sciences, Wenzhou Medical University, Wenzhou, China; ^2^ Department of Gynecologic Oncology, Women’s Hospital, School of Medicine, Zhejiang University, Hangzhou, China; ^3^ First Clinical College, Wenzhou Medical University, Wenzhou, China; ^4^ Department of Gynecologic Oncology, Wenzhou Central Hospital, Wenzhou, China; ^5^ Department of Obstetrics and Gynecology, The First Affiliated Hospital, Wenzhou Medical University, Wenzhou, China; ^6^ Department of Obstetrics and Gynecology, The Second Affiliated Hospital and Yuying Children’s Hospital of Wenzhou Medical University, Wenzhou, China

**Keywords:** serous ovarian carcinoma, RNA modification writers, lncRNA, prognosis, immune microenvironment

## Abstract

Serous ovarian carcinoma (SOC) is a gynecological malignancy with high mortality rates. Currently, there is a lack of reliable biomarkers for accurate SOC patient prognosis. Here, we analyzed SOC RNA-Seq data from The Cancer Genome Atlas (TCGA) to identify prognostic biomarkers. Through the pearson correlation analysis, univariate Cox regression analysis, and LASSO-penalized Cox regression analysis, we identified nine lncRNAs significantly associated with four types of RNA modification writers (m^6^A, m^1^A, APA, and A-I) and with the prognosis of SOC patients (*P <*0.05). Six writer-related lncRNAs were ultimately selected following multivariate Cox analysis. We established a risk prediction model based on these six lncRNAs and evaluated its prognostic value in multiple groups (training set, testing set, and entire set). Our risk prediction model could effectively predict the prognosis of SOC patients with different clinical characteristics and their responses to immunotherapy. Lastly, we validated the predictive reliability and sensitivity of the lncRNA-based model *via* a nomogram. This study explored the association between RNA modification writer-related lncRNAs and SOC prognosis, providing a potential complement for the clinical management of SOC patients.

## Introduction

Ovarian cancer (OC) is among the deadliest gynecological malignancies. In 2020, there were more than 313,000 new cases of OC globally, in addition to more than 207,000 deaths, and these numbers continue to rise (1, 2). Serous ovarian carcinoma (SOC) accounts for approximately 75% of the OC cases, representing the most common histological OC subtype (3). Approximately 70% of OC patients already have advanced-stage disease at the time of diagnosis, and a large proportion experience disease relapse due to the lack of effective screening tools for early diagnosis (4). Although treatment methods have improved recently, the prognosis remains far from optimal (5). Due to the limitations of available SOC treatment, there is an urgent need for the identification of sensitive prognostic markers and the introduction of new predictive models for treatment response to guide personalized therapy.

RNA modification is a key epigenetic process that regulates post-transcriptional gene expression (6), with more than 170 types of post-transcriptional RNA modifications identified at present, namely, N6-methyladenosine (m^6^A), N1-methyladenosine (m^1^A), alternative polyadenylation (APA), adenosine-to-inosine (A-I), and others (7). Adenine is the most heavily modified nucleotide in RNA (8, 9). Currently, research on adenine modifications is mainly focused on m^6^A, m^1^A, APA, and A-I. At present, known m^6^A writers include methyltransferase-like protein 3/14 (METTL3/14), Wilms’ tumor-associated protein (WTAP), RNA-binding motif protein 15/15B (RBM15/15B), zinc finger CCCH-Type containing 13 (ZC3H13), and KIAA1429 (VIRMA, vir-like m^6^A methyltransferase associated) (10); m^1^A writers include tRNA methyltransferase 6/61A/61B/10C (TRMT6/61A/61B/10C) (11); APA writers include cleavage and polyadenylation specificity factor 1–4 (CPSF1–4), cleavage stimulation factor 1–3 (CSTF 1–3), cleavage factor I (CFI), PCF11 (protein 1 of CFI), cleavage factor polyribonucleotide kinase subunit 1 (CLP1), and nuclear poly(A)-binding protein 1 (PABPN1) (12); A-I writers include adenosine deaminases acting on RNA (ADARs, such as ADAR, ADARB1, and ADARB2) (13). Multiple studies have shown that these four RNA modifications and their respective writer enzymes play an important role in the incidence and development of various cancer types, including SOC (8, 10). Through the analysis of 11,552 samples derived from 39 tissue and cell types, Ali et al. discovered that changes in the mitochondrial RNA N1-methyladenosine and N1-methylguanine (m^1^A/G) modification levels affected mutations in nuclear DNA, thereby promoting the progression of breast cancer (14). Bi et al. found that METTL3 mediated the maturation of microRNA-126-5p through m^6^A modification, resulting in miRNA binding to phosphatase and tensin homolog and, thereby, activating the P13K/Akt/mTOR pathway, which in turn promoted OC incidence and progression (15). Loss of CPSF1 suppressed OC cell viability, induced cell cycle arrest in the G0/G1 phase and promoted cellular apoptosis (16). Amin et al. found that ADAR upregulation is an independent predictor of lung adenocarcinoma relapse and that ADAR increases FAK expression by catalyzing the A-I modification on RNA, thus promoting the migration and invasion of lung adenocarcinoma cells (17). Taken together, the dysregulation of multiple types of RNA modifications may contribute to the development of cancer. Additionally, interactions have been reported between different modifications. Xiang et al. showed that m^6^A modifications could suppress the binding of A-I writer ADAR to RNA, downregulating of A-I modification levels in methylated transcripts (18). Dai et al. used an unbiased quantitative proteomic method and confirmed that m^6^A reader YTH domain-containing family 2 can bind to m^1^A with low affinity, accelerating the degradation of m^1^A-modified transcripts (19), thus suggesting functional crosstalk between m^6^A and m^1^A modifications. Molinie et al. found that the distribution of m^6^A modification on transcripts may be related to that of APA modification sites (20). Taken together, these findings indicate that different types of adenine modifications, particularly m^6^A, m^1^A, APA, and A-I, may have complicated regulatory networks (9). There is growing evidence that RNA modification writers play an essential role in inflammation and innate immunity by interacting with various writers (9). Chen et al. revealed crosstalk among m^6^A, m^1^A, APA, and A-I writers in colorectal cancer and demonstrated their potential therapeutic value in colorectal cancer (9). However, no studies have explored the combined effects of m^6^A, m^1^A, APA, and A-I modifications on the pathogenesis and treatment response of SOC. Hence, we focused our research on the writer enzymes of these four RNA modifications (m^6^A, m^1^A, APA, and A-I).

Long non-coding RNAs (lncRNAs) are transcripts with a length of more than 200 nucleotides that have no or only limited protein-coding ability and influence cancer progression through their interaction with DNA, protein, or RNA, to regulate signal transduction (21, 22). Multiple studies have shown that m^6^A writers are involved in the regulation of the biological functions of lncRNAs (22). For instance, Xue et al. found that METTL3 enhanced the stability of the lncRNA ABHD11-AS1 by catalyzing its m^6^A modification, thus promoting the proliferation of non-small cell lung cancer (23). With respect to other adenine RNA modification types (such as m^1^A and A-I), few studies have explored the roles of their writer enzymes in lncRNA regulation. Most available research only used sequencing technology and bioinformatic analysis to preliminarily explore the distribution of these modifications on lncRNAs in cancer cells (24–26). Interestingly, studies have also shown that lncRNA could influence the function of RNA modifications. For example, Zhu et al. found that the RNA-binding regulatory peptide encoded by the lncRNA LINC00266-1 is the regulatory subunit of insulin-like growth factor 2 mRNA-binding protein 1 (IGF2BP1). Further, this regulatory subunit regulated the recognition of m^6^A RNA by IGF2BP1 and mediated the stabilization of c-Myc and other mRNA transcripts, thereby promoting tumor incidence and development (27). There are still relatively few studies on lncRNAs related to RNA modification writers in SOC. A comprehensive understanding of the effects of writer-related lncRNAs on the prognosis and immune response in SOC will help us better understand the SOC tumor microenvironment and thus guide immunotherapy strategies.

Previous studies have validated lncRNAs related to RNA modification writers in multiple cancers such as breast cancer (28), bladder cancer (29), and lung adenocarcinoma (30), but not in SOC. Here, we screened for lncRNAs related to RNA modification writers based on the transcriptomic data of SOC patients obtained from The Cancer Genome Atlas (TCGA) database, with the aim to identify prognostic lncRNA biomarkers. We obtained six lncRNAs related to the four types of RNA modification writers, which were significantly associated with the prognosis of SOC. Subsequently, we established a prognostic risk score model (m^6^A/m^1^A/A-I/APA-LPR) based on these six lncRNAs and validated its prognostic accuracy for SOC. Finally, we explored the correlation between our risk model and the tumor microenvironment as well as immunotherapy response. The current study provides potential biomarkers for SOC prognosis and management.

## Materials and Methods

### Gene Expression Profiles and Clinical Data of Patients With SOC

RNA sequencing and mutation data of patients with SOC (N = 375) from the TCGA database were downloaded using “TCGAbiolinks” (R package), and the corresponding clinical information was downloaded from the GDC database (https://cancergenome.nih.gov/). SOC patients with missing survival information were excluded. Patients were randomly separated into two cohorts at a 4:6 ratio, named the training set and the testing set, respectively, for the establishment and validation of the risk model. The total TCGA patient dataset is referred to as the “entire set”.

The RNA modification writers consisted of seven m^6^A modification enzymes (METTL3, METTL14, WTAP, RBM15, RBM15B, ZC3H13, and KIAA1429), four m^1^A modification enzymes (TRMT61A, TRMT61B, TRMT10C, and TRMT6), 12 APA modification enzymes (CPSF1-4, CSTF1/2/3, PCF11, CFI, CLP1, NUDT21, and PABPN1), and three A-I modification enzymes (ADAR, ADARB1, and ADARB2). The expression profiles for lncRNA, mRNA, and adenosine RNA modification writer genes were separately acquired for subsequent analyses.

### Correlation Analysis

We screened four types of RNA modification writer-related lncRNAs *via* pearson correlation analysis in entire set using the “rcorr” function from “Hmisc” (R package), with the criteria of |Pearson R| >0.3 and *P <*0.001 (30).

### Reverse Transcription Quantitative Polymerase Chain Reaction of m^6^A/m^1^A/A-I/APA-LPR Model-Associated lncRNAs

The human OC cell lines CAOV3, OVCAR3, and SKOV3 were purchased from American Type Cell Culture (ATCC, Manassas, VT, USA), and A2780 was purchased from Sigma-Aldrich (Cat#93112519, St Louis, Missouri, USA). Cisplatin-resistant cell lines (SKOV3-CIS and A2780-CIS) were established in our lab. The normal ovarian epithelial cell line IOSE-80 was purchased from MeisenCTCC (Zhejiang Meisen Cell Technology Co., Ltd., Hangzhou, China). All cell lines were cultured in Dulbecco’s modified Eagle’s medium (Gibco, Thermo Fisher Scientific Inc., Thermo Fisher Scientific Inc., Waltham, MA, USA) containing 10% fetal bovine serum at 37°C with 5% CO_2_. For RNA purification, the isolated cells were lysed in TRIzol reagent (Invitrogen Life Technologies, Grand Island, NY, USA). The extracted RNA was further digested using DNase I (Invitrogen, Waltham, MA, USA) to remove residual DNA and subsequently separated from each sample using TRIzol reagent/RNeasy Mini kit (Qiagen, Hilden, Germany). The total extracted RNA was stored at −80°C for future use.

The lncRNA expression levels in both the OC cell lines and normal ovarian epithelial cell lines were measured by performing a reverse transcription quantitative polymerase chain reaction (qRT-PCR) using an Applied Biosystems QuantStudioTM 6 real-time PCR instrument (Thermo Fisher Scientific Inc., Waltham, MA, USA). All qRT-PCR experiments were performed using the QuantiNova SYBR Green PCR kit (Qiagen, Hilden, Germany). For each reaction, 1 µl of diluted cDNA was mixed with 18.2 µl of 1× SYBR Green PCR Master Mix. A final volume of 20 µl was achieved by adding 0.4 µl each of the forward and reverse primers (10 µmol). The conditions for PCR amplification were as follows: 95°C for 5 min, followed by 40 cycles each of 95°C for 10 s and 60°C for 30 s. All samples were tested in triplicate. The data were analyzed using the comparative threshold cycle (Ct) method. GAPDH was used as the control, and the relative quantification of lncRNAs in cells was calculated using the following equation: amount of target = 2^−ΔCt^, where ΔCt = Ct_lncRNA_ − Ct_GAPDH_. The gene-specific primers for lncRNA and GAPDH used for qRT-PCR are listed in **Supplementary Table 1**.

### m^6^A/m^1^A/A-I/APA-LPR Model Construction and Validation

As previously reported (30, 31), the training set was used to construct a writer-related lncRNA model, and the lncRNAs were selected based on univariate Cox regression and LASSO Cox (10-fold cross-validation) analyses using “survival” and “glmnet” (R packages) and visualized *via* “ROCR,” “survminer,” “ComplexHeatmap,” and “ggplot2” (R packages). The risk score was calculated as the sum of the prognostic coefficients multiplied by the expression profiles of writer-related lncRNAs. Six writer-related lncRNAs (AC142528.1, PCAT29, RP11-508M8.1, MYCNOS, RP11-327F22.2, and RP11-665C16.5) were identified for establishing the risk model. The following formula was used to calculate the risk score: m^6^A/m^1^A/A-I/APA score = h0(t) × [0.010174 × expression(RP11-508M8.1) + 0.003821 × expression(RP11-665C16.5) − 0.136630 × expression(AC142528.1) − 0.081020 × expression(MYCNOS) − 0.028803 × expression(PCAT29) − 0.016343 × expression(RP11-327F22.2)], where h0(t) is the baseline risk of m^6^A/m^1^A/A-I/APA score when all variables are 0, as per a previous report (30). Patients were divided into low- and high-risk groups based on their risk scores in each cohort (training, testing, and entire sets). The latter two sets were used to validate the prognostic value of our established model, with the median risk score obtained in the training set used as the cut-off value.

### Principal Component Analysis

Principal component analysis (PCA) was used for reducing the effective dimensionality, identifying the model, grouping *via* the “prcomp” function in R, and visualized using “scatterplot3d” (R package).

### Mutation Analysis

The mutation profile was analyzed and visualized using “maftools” (R package).

### Functional and Pathway Enrichment Analyses and Exploration of the Risk Model for Immunotherapy Response Prediction

The immune scores of SOC patients were downloaded from the ESTIMATE database (Estimation of STromal and Immune cells in MAlignant Tumor tissues using Expression data; https://bioinformatics.mdanderson.org/estimate/). Immune-related gene sets used for GESA in this study were downloaded from the MSigDB database (https://www.gsea-msigdb.org/gsea/index.jsp), including “IMMUNE RESPONSE.gmt,” “29immunesets.gmt,” “h.all.v7.4.symbols.gmt,” “c2.cp.kegg.v7.4.symbols.gmt,” and analyzed using “GSVA” (R package). We used the TIDE algorithm (http://tide.dfci.harvard.edu) to predict the likelihood of an immunotherapeutic response. Therapeutic responses to various drugs were predicted using “oncoPredict” (R package). LncRNA-related drugs were predicted using the LncMAP database (http://bio-bigdata.hrbmu.edu.cn/LncMAP/) and visualized *via* Cytoscape (version 3.9.0, http://www.cytoscape.org/).

### Nomogram Construction and Evaluation

Nomogram and calibration curves were constructed and visualized using the “survival” and “rms” (R packages). Receiver operating characteristic (ROC) curves were analyzed and visualized using “ROCR,” “pROC,” and “timeROC” (R packages).

### Statistical Analysis

Continuous variables were analyzed using Student’s t-tests or non-parametric Wilcoxon tests. Prognostic analyses were performed *via* Kaplan–Meier and Cox regression analyses using “survminer” and “survival” (R packages). R 4.0.1 (http://www.r-project.org/) was used to analyze all data. The results with *P <*0.05 were considered statistically significant.

## Results

### Identification of lncRNAs Related to RNA Modification Writers in Patients With SOC

**Figure 1:**
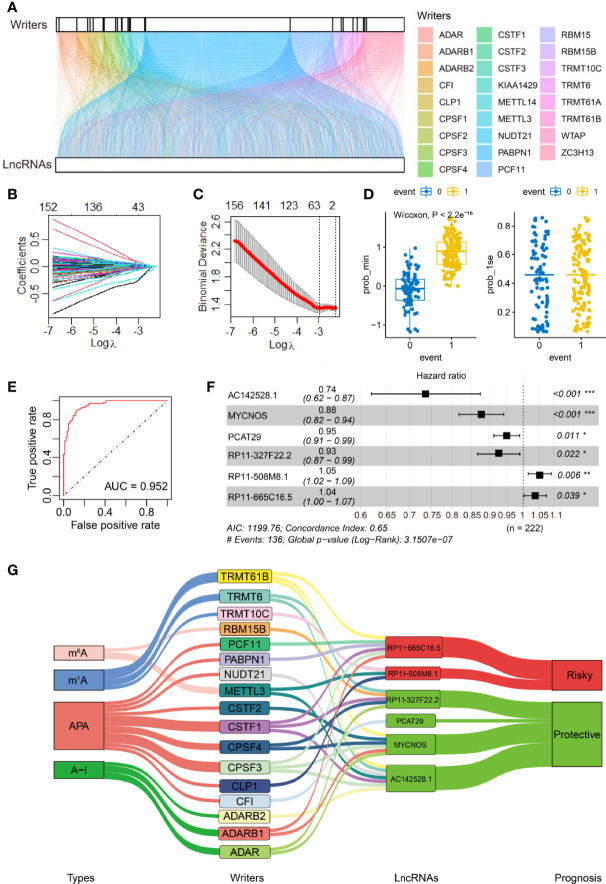
Identification of RNA adenosine modification writer-related lncRNAs and establishment of the lncRNA-based risk model. **(A)** Alluvial diagram for 26 writer genes and writer-related lncRNAs. **(B)** The LASSO coefficient profile of OS-related lncRNAs was drawn *via* 10-fold cross-validation. **(C)** The tuning parameters (log λ) of OS-related proteins were selected to cross-verify the error curve. Of the two dotted lines in the figure, the left is λ Min, and the right is λ 1se. λ Min is the value of λ that gives the minimum mean cross-validated error, whereas the other λ saved is λ 1se, which gives the most regularized model such that error is within one standard error of the minimum. **(D)** Self-prediction based on the minimal criterion and 1se criterion (0 and 1 represent the states where events are predicted to occur and not to occur, respectively, according to the model). **(E)** ROC curves of the model *via* internal validation. **(F)** Multivariate Cox regression analysis yielded six independent prognostic lncRNAs. PR11-508M8.1 and PR11-665C16.5 were risk factors, and the other four lncRNAs were protective factors for SOC. *P <0.05, **P <0.01, ^***^ *P <*0.001. **(G)** Relational Sankey diagram for significant correlations between 17 writer genes and six prognostic writer-related lncRNAs.

We have summarized the process of biomarker identification in a flowchart (**Supplementary Figure 1**). We obtained the full transcriptome data of 375 SOC patients from the TCGA database. We identified 15,900 lncRNAs and 26 writer genes (wirters) (**Figure 1A**). We screened 2,460 writer-related lncRNAs through pearson correlation analysis (|R| >0.3 and *P <*0.001, **Supplementary Table 2**).

### Establishment of a Risk Model Based on lncRNAs Related to RNA Modification Writers in SOC Patients

First, 163 writer-related lncRNAs were significantly correlated with OC survival based on Cox univariate analysis in our training set (*P <*0.05, **Supplementary Table 3**). We then performed LASSO Cox analysis to further narrow down prognosis-related lncRNAs. The coefficients of candidate lncRNAs were obtained (**Figure 1B**), and 40 writer-related lncRNAs were selected *via* the λ minimization method (**Figure 1C**). Concurrently, we carried out a model self-rating, which indicated that the lncRNA-based risk model could easily differentiate between patients based on survival status (**Figure 1D**). An ROC analysis was performed to evaluate the prognostic value of candidate writer-related lncRNAs. The area under the ROC curve was 0.952, which suggested that these lncRNAs could effectively predict prognosis (**Figure 1E**). Next, six writer-related lncRNAs were obtained *via* multivariate Cox analysis (**Figure 1F**). The expression of these six lncRNAs was visualized in this study and checked in the TANRIC database (32) (**Supplementary Figures 2A, B**). Based on four lncRNA-databases, namely, Lnc2Cancer 3.0 (33), LncCAR (34), Immlnc (35), and LncMAP (36), we also found that these lncRNAs were expressed in OC. Additionally, we performed qRT-PCR to detect and validate the expression of the six lncRNAs in six OC cell lines and one ovarian epithelial cell line (**Supplementary Figure 2C**).

These lncRNAs were independently correlated with OC survival (**Figure 1F**). We established a prognostic risk model based on the expression profiles and the regression coefficients of these lncRNAs in the training set, and the C-index of our risk model was 0.646 (se = 0.024) (**Figure 1F**). We also visualized the significant association between lncRNAs and the 17 associated writers (out of the above mentioned 26 writers). We found that 2/17 (m^6^A), 3/17 (m^1^A), 9/17 (APA), and 3/17 (A-I) writers were significantly associated with these candidate lncRNAs (**Figure 1G**). Of the six candidate lncRNAs, AC142528.1, MYCNOS, PCAT29, and RP11-327F22.2 were protective factors in SOC (hazard ratio (HR) <1), while PR11-508M8.1 and PR11-665C16.5 were risk factors (HR >1) (**Figure 1G**; **Supplementary Figure 3**).

**Figure 2:**
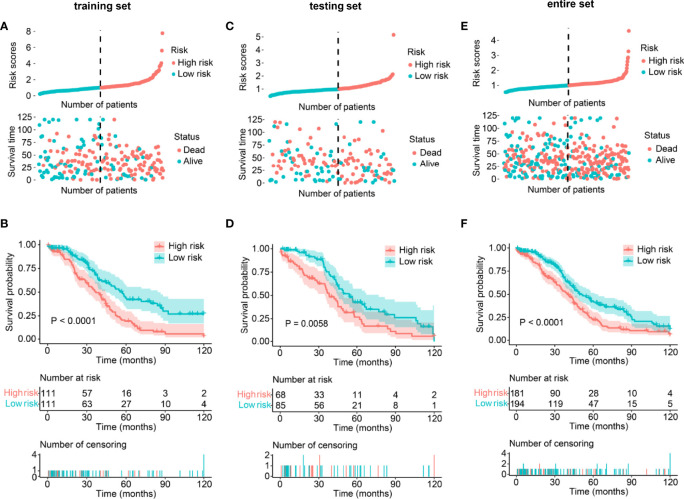
Validation of the lncRNA-based prognostic risk model in the training set, testing set, and entire set. **(A, C, E)** Distribution of risk score and survival status between low/high-risk SOC patients in the training set **(A)**, testing set **(C)**, and entire set **(E)**. The blue color represents patients with a low risk score, and the red color represents patients with a high risk score. Distribution of risk score based on the writer-related lncRNA model (Upper panel). Survival status and survival time between the high- and low-risk subgroups (Lower panel). **(B, D, F)** Kaplan–Meier survival analysis between low- and high-risk subgroups of patients in the training set **(B)**, testing set **(D)**, and entire set **(F)**.

Risk scores were calculated for the training set, and patients were then grouped into low- and high-risk groups with the median risk score (0.99681) as a cutoff value. The risk score distribution, survival time, survival status, and expression level of the six writer-related lncRNAs for each patient in the training set are shown in **Supplementary Figure 3**. Survival analysis indicated that the overall survival (OS) of the patients in the low-risk group was greater than that of the patients in the high-risk group (*P <*0.0001, **Figure 2A**).

### Validation of Our Risk Model in SOC Patients

To validate the prognostic value of the above-established risk model, risk scores were calculated for every patient in the testing and entire sets. Patients were again divided into low- and high-risk groups. The distribution of risk scores, survival status, and survival time was visualized (**Figure 2**). As expected, Kaplan–Meier survival analysis also suggested that patients with a high-risk score had a worse OS than those with low-risk scores (*P_testing set_* = 0.0058, *P_entire set <_* 0.0001, **Figures 2B, C**). The above results indicated that the risk model could be used to predict SOC prognosis accurately.

**Figure 3:**
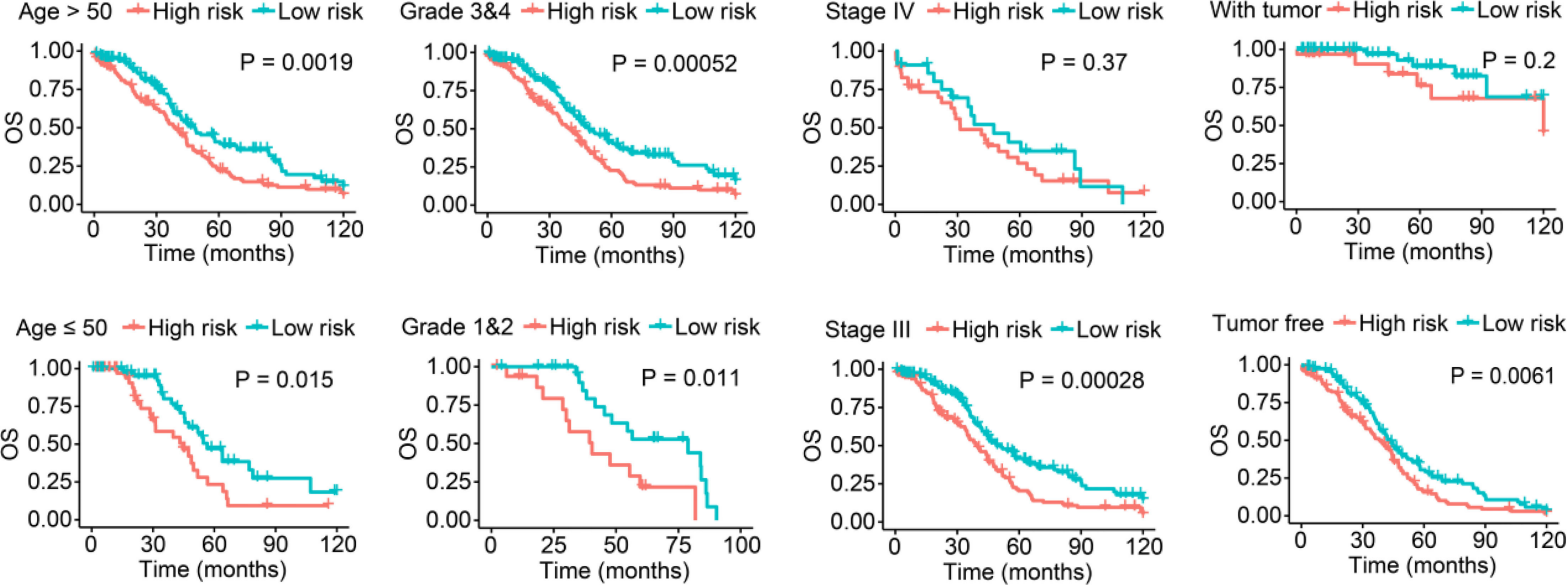
Survival analysis stratified by age, tumor grade, FIGO stage, and tumor status between the low- and high-risk groups in the entire set.

Additionally, we stratified low- and high-risk patients in the entire set according to their clinicopathological features and analyzed the differences in OS. In the subgroups classified by age and tumor grade, the OS of low-risk patients was significantly longer than that of high-risk patients (**Figure 3**). Moreover, although there was no statistically significant difference, we found discrepancies in the OS between low- and high-risk SOC patients with FIGO stage IV or with tumors (**Figure 3**).

### Principal Component Analysis Further Verified the Prognostic Value of our m^6^A/m^1^A/A-I/APA-LPR Model

**Figure 4:**
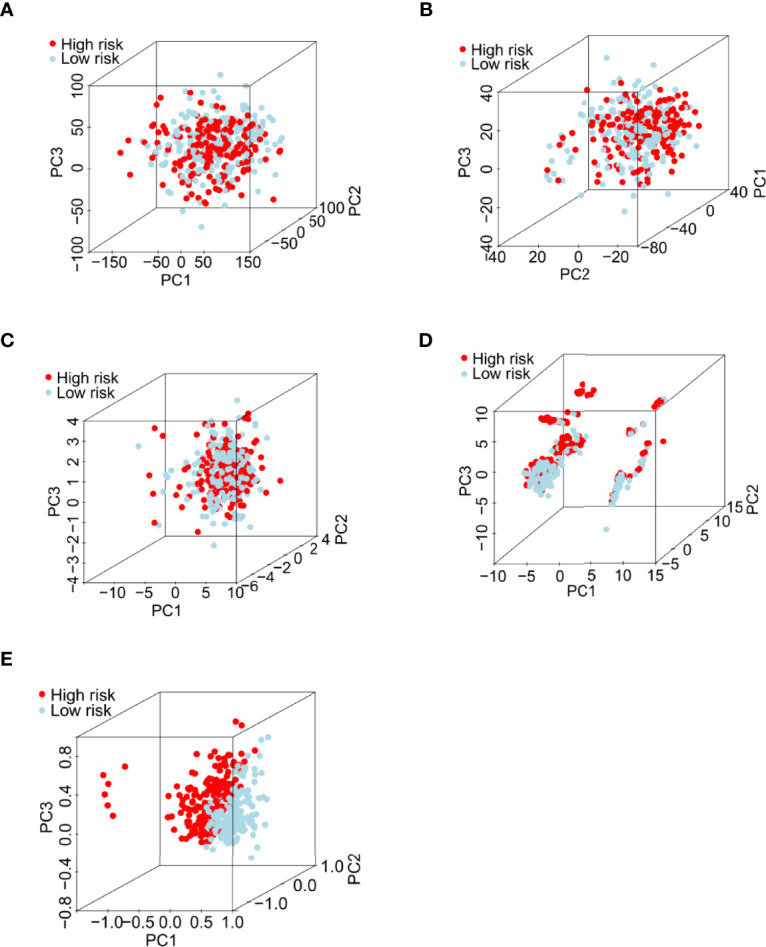
Principal component analysis between the low- and high-risk groups in the entire set. **(A)** All RNA-seq data from the TCGA database. **(B)** Expression profiles of all coding genes. **(C)** Expression profiles of 26 writer-related genes. **(D)** Expression profiles of six writer-related lncRNAs. **(E)** Risk model based on the profiles of the six writer-related lncRNAs.

PCA was performed to evaluate the ability of our risk model to discriminate between low- and high-risk patients based on gene expression profiles of 1) all RNA-seq data (**Figure 4A**); 2) coding genes (**Figure 4B**); 3) 26 writer genes (**Figure 4C**); 4) six writer-related lncRNAs (**Figure 4D**); and 5) risk model classified by the expression profiles of the six writer-related lncRNAs (**Figure 4E**). The gene expression profiles of the six writer-related lncRNAs could effectively distinguish patients (**Figure 4D**), especially for the risk model (**Figure 4E**). However, we did not obtain similar results based on other data (**Figures 4A–C**). These findings suggest that the model established based on writer-related lncRNAs could be a potential prognostic signature.

### The Prognostic Value of m^6^A/m^1^A/A-I/APA-LPR Was Greater Than That of TP53 Mutation Status

**Figure 5:**
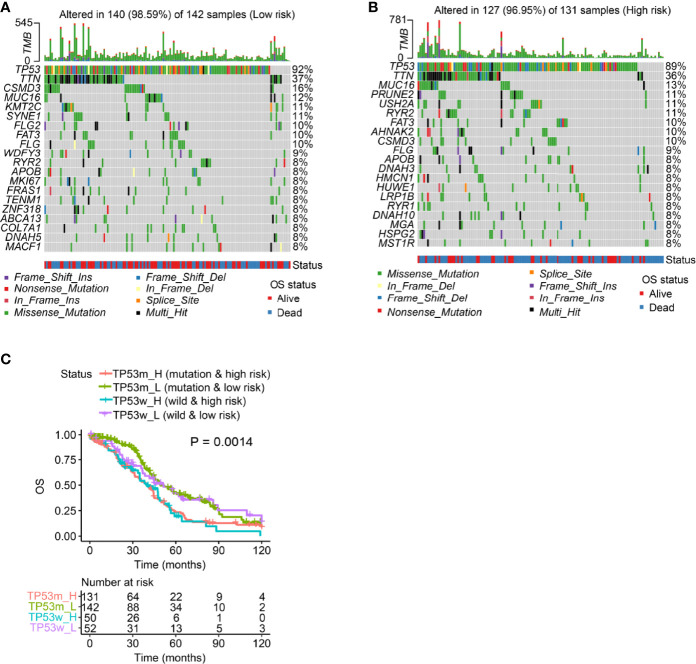
Mutation analysis using the risk model in the entire set. **(A, B)** Waterfall plot displays mutation information of the genes with high mutation frequencies in the patients with low-risk scores **(A)** and those with high-risk scores **(B)**. **(C)** Overall survival analysis of patients classified according to the m^6^A/m^1^A/A-I/APA-LPR score and *TP53* mutation status in the entire set.

We visualized the top 20 most frequently mutated genes in the low- and high-risk patient groups, and our results indicated that TP53 had the highest mutation frequency in both groups (low-risk: 92%; high-risk: 89%; **Figures 5A, B**). TP53 mutations are present in various human cancers (pancreatic adenocarcinoma, liver hepatocellular carcinoma, chromophobe renal cell carcinoma, acute myeloid leukemia, thymoma, etc.) and represent potential prognostic markers (37). Thus, we explored whether the m^6^A/m^1^A/A-I/APA-LPR model could predict OS better than TP53 mutation status. Surprisingly, the survival results of high-/low-risk patients with TP53 mutation were similar to those of high-/low-risk patients with wild-type TP53, indicating that the TP53 mutation status failed to prognostically distinguish SOC patients. Interestingly, the low-risk patients had an apparently longer OS than those with high-risk scores, regardless of TP53 mutation status (**Figure 5C**). These results indicated that our risk model was a better predictor of SOC prognosis than TP53 mutation status.

### Stratification Analysis of the m^6^A/m^1^A/A-I/APA-LPR Model With Regard to Tumor Immune Microenvironment and Cancer Immunotherapy Response

**Figure 6:**
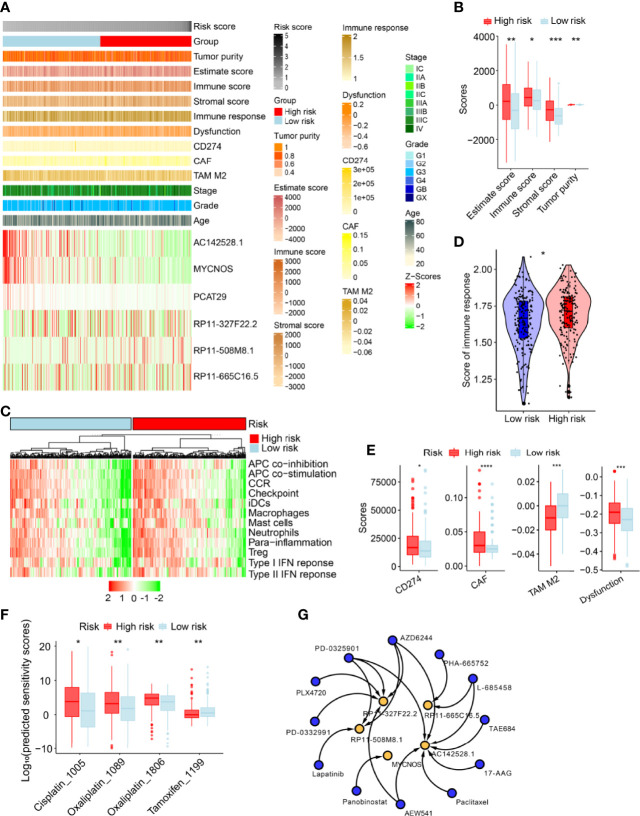
Estimation of immune-related factors using the risk model in the entire set. **(A)** Heatmap of associations between the expression levels of the six m^6^A/m^1^A/A-I/APA-related lncRNAs and clinicopathological features. **(B)** The differences in stromal and immune cell scores between low- and high-risk patients were analyzed. **(C)** The indicated standards of the immunity index for each patient were visualized *via* heatmaps, with red representing high expression, and green representing relatively low expression. **(D)** The differences in immune response between low- and high-risk SOC patients. **(E)** Estimation of cancer immunotherapy response. **(F)** Differences in sensitivity against clinical applied drugs. **(G)** Twelve potential drugs (blue) were screened based on interactions of the RNA adenosine modification writer-related lncRNAs (yellow) in the drug–lncRNA module of LncMAP database. Only statistically significant results are shown (*P <*0.05). *P <0.01, **P <0.05, ***P <0.001, ****P <0.0001.

We performed subsequent analyses (**Figure 6A**) to explore differences in tumor immune microenvironment between low- and high-risk patients. As expected, SOC patients with high-risk scores had higher immune and stromal cell scores than low-risk patients did. Furthermore, the tumor purity of high-risk patients was higher (**Figure 6B**). High-risk patients exhibited high expression of immune factors (such as CCR and APC co-inhibition) and tumor-infiltrating immune cells (such as interdigitating dendritic cells, macrophages, mast cells, and neutrophils) (**Figure 6C**). We then analyzed the difference in immune responses between low- and high-risk SOC patients, with the latter having higher immune response scores (**Figure 6D**). To explore the molecular mechanisms underlying SOC progression, we performed hallmark gene signature and Kyoto Encyclopedia of Genes and Genomes (KEGG) pathway enrichment analyses, which revealed significant discrepancies in various immune-related biological processes between the low- and high-risk groups. For example, the high-risk group had higher scores for IL2-STAT5 signaling, IL6-JAK-STAT3 signaling, and B-cell receptor signaling than the low-risk group (**Supplementary Figures 4A, B**). Along with the above-described results, we explored the correlation between the risk model and immunotherapy response. As expected, we found that low-risk patients were more likely to respond to immunotherapy than high-risk ones, indicating that this risk model based on immune indexes (i.e., cluster of differentiation 274/programmed cell death ligand 1 (CD274/PD-L1) carcinoma-associated fibroblasts (CAFs)) might serve as an indicator for predicting tumor immune dysfunction and exclusion (TIDE), excluding tumor-associated macrophages (**Figure 6E**).

### Identification of Novel Potential Drugs for the Treatment of Patients With High m^6^A/m^1^A/A-I/APA-LPR Risk Scores

We further evaluated the therapeutic response for every patient in the entire set based on the half-maximal inhibitory concentration (IC50) of various drugs available in the Genomics of Drug Sensitivity in Cancer (GDSC) database. Therapeutic score prediction analysis revealed that 35 drugs had significantly different efficacy between the two groups (**Supplementary Table 4**). As expected, low-risk SOC patients were more sensitive to Cisplatin_1005 and Oxalipatin_1089/1086, but not to Tamoxifen_1199 (**Figure 6F**). We performed lncRNA–drug prediction analysis, as described in the *Materials and Methods* section. Predicted were 120 paired lncRNA-drug interactions, which included the five lncRNAs (AC142528.1, MYCNOS, RP11-327F22.2, PR11-508M8.1, and PR11-665C16.5) and 24 drugs (**Supplementary Table 5**). We screened and constructed a network of 18 lncRNA–drug pairs (*P <*0.05) out of the 120 lncRNA–drug pair interactions (**Figure 6G**).

### Evaluation of the lncRNA-Based Prognostic Risk Score Model Together With Clinical Features in SOC Patients

Combining the risk score, FIGO stage, grade, and age of patients, we conducted univariate and multivariate Cox regression analyses to evaluate prognostic value in SOC patients. Only the risk score was an independent factor for OS (**Supplementary Figure 5A**, *P <*0.001). In univariate cox regression analysis, the risk score had an HR and a 95% confidence interval (CI) of 1.57 and 1.25–1.97, respectively. In multivariate cox regression analysis, the HR was 1.54, and the 95% CI was 1.22–1.94. These results highlighted our risk model as the only independent prognostic factor in SOC patients (**Supplementary Figure 5A**). The area under the ROC curve (AUC) was assessed, with the risk score model showing a larger AUC than other clinicopathological characteristics (AUC_Risk model_ = 0.638, AUC_FIGOstage_ = 0.566, AUC_Grade_ = 0.499, AUC_Age_ = 0.561; **Supplementary Figure 5B**). The m^6^A/m^1^A/A-I/APA-LPR risk model also performed well at differentiating follow-up time, and its concordance index was larger than that of other clinical factors over time (**Supplementary Figures 5C, D**). These results indicated that the prognostic capacity of m^6^A/m^1^A/A-I/APA-LPR in SOC patients was robust.

### Establishment and Evaluation of a Prognostic Risk Score-Based Nomogram

**Figure 7:**
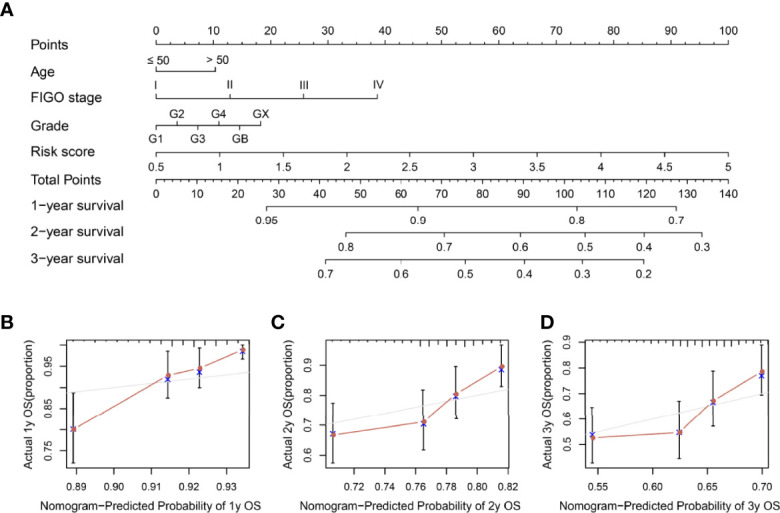
Nomogram construction and visualization. **(A)** A nomogram constructed using risk score and clinical characteristics in SOC patients within 1-, 2-, and 3-year OS data. **(B–D)** Calibration plots of actual and predicted 1-, 2-, and 3-year OS in the entire set.

To further evaluate the potential of our risk model in predicting SOC patient outcomes, we established a risk score-based nomogram. More specifically, the nomogram included clinical characteristics and the risk model. We then used it to predict the 1-, 2-, and 3-year OS. In comparison with clinical characteristics alone, the nomogram exhibited greater predictive ability (**Figure 7A**). Moreover, calibration analysis revealed a coherence between the prediction curves of the risk model and the actual 1-, 2-, and 3-year survival curves (**Figures 7B–D**), further highlighting the prognostic accuracy of the nomogram.

## Discussion

Studies have shown that the interaction of different writers mediates abnormal RNA modifications, which promote tumor proliferation, migration, and invasion, as well as immune regulation (8, 38). Through their regulatory effects on gene expression and signaling pathways, lncRNAs influence tumor progression and even contribute to treatment resistance in various tumors, including OC (39, 40). Numerous studies have explored the significance of RNA modifications, especially the association between m^6^A and lncRNA, in different tumors. METTL3 mediates the m^6^A modification of the lncRNA THAP7-AS1, enhancing its expression and thereby, promoting the interaction between its nuclear localization signal and importin α1. This allows the CUL4B protein to enter the nucleus and inhibit miR-22-3p and miR-320a transcription, thus promoting gastric tumorigenesis (41). The stability of lncRNA RMRP is enhanced through m^6^A modification, regulating the TGFBR1/SMAD2/SMAD3 pathway and the proliferation and progression of non-small cell lung cancer (42). While these studies highlight the role of RNA modification writer-related lncRNAs in human cancers, the study of these lncRNAs is still in its infancy (43–45). We believe that exploring the interactions between lncRNAs and RNA modification writers will lead to the identification of new prognostic markers or therapeutic targets for malignant tumors.

Through bioinformatics analysis of SOC RNA-Seq data from the TCGA database, we obtained six RNA modification writer-related lncRNAs (AC142528.1, MYCNOS, PCAT29, PR11-327F22.2, PR11-508M8.1, and PR11-665C16.5) that were significantly related to the prognosis of SOC (**Supplementary Figure 5A** and **Figure 1F**). Based on the expression profiles of these lncRNAs and their regression coefficients, we established the m^6^A/m^1^A/A-I/APA-LPR prognostic model. Among the six lncRNAs, MYCNOS promotes tumorigenesis in various cancers. It is upregulated in glioblastoma where it might promote tumor cell proliferation *via* the MYCNOS/miR-216B/FOXM1 axis (46). Additionally, MYCNOS was closely related to the poor prognosis of hepatocellular carcinoma based on bioinformatics analysis (47). Although available research on MYCNOS is still limited, its biological function in SOC is yet to be explored, considering that some lncRNAs play opposite roles in different cancer types, as previously described for metastasis-associated lung adenocarcinoma transcript 1 (MALAT1) (48). Various studies have shown that MALAT1 exerts tumor-promoting effects in several cancers, including non-small cell lung cancer, osteosarcoma, cervical cancer, and pancreatic cancer (49). However, MALAT1 was downregulated in glioma and endometrioid endometrial carcinoma, where it exerted tumor-suppressive effects (50, 51). Recently, MALAT1 was reported to bind and inactivate TEAD (TEA/ATTS domain), inhibiting breast cancer metastasis in transgenic, xenograft, and syngeneic mouse models (52). Interestingly, previous bioinformatics analysis studies suggested that MALAT1 was associated with a poor prognosis of breast cancer (53, 54). These findings highlight the complexity of lncRNA involvement in different cancers. Our group established a model for predicting SOC prognosis and immunotherapy response based on m^6^A effector-related lncRNAs (unpublished data). Similarly, we identified MYCNOS as a protective factor in SOC, with a potentially important role in its incidence and development. Nevertheless, whether MYCNOS exerts a tumor-suppressive or tumor-promoting effect in SOC remains to be further investigated. PCAT29 acts as a tumor suppressor and downregulates the proliferation and migration of prostate cancer cells (55). Moreover, Bao et al. found that PCAT29 was expressed in OC and the positive rate of PCAT29 was 82/116; they also identified PCAT29 as a signature associated with prognosis in pan-cancer (including OC) (56). PR11-508M8.1 was proposed as a biomarker for predicting the risk of papillary thyroid carcinoma relapse (57). Data regarding the cancer-related functions of the remaining three lncRNAs in our model, namely, AC142528.1, PR11-327F22.2, and PR11-665C16.5, are scarce. Validation in our training set (n = 153) and the entire set (n = 375) confirmed the prognostic value of the lncRNA-based model in SOC. To further explore the significance of our model with respect to the tumor microenvironment, we analyzed the differences in the expression of CD274/PD-L1 as well as the infiltration of CAFs and tumor-associated macrophages (TAMs) in high-risk and low-risk patient groups. The low-risk patient group had lower CD274/PD-L1 and CAF scores than the high-risk group, while the TAM score was greater than in the high-risk group. Research has shown that various cancers use the PD-L1 and programmed cell death-1 (PD-1) immune checkpoints to evade T cell immunity, and blocking their interaction has significant anti-tumor effects in patients with advanced cancer (58). Furthermore, the combination of PARP inhibitors with anti-PD-1/PD-L1 drugs was reported to have a synergistic anti-OC activity (59). CAFs are activated by various cytokines, which promote tumorigenesis, accelerate tumor invasion and metastasis, induce angiogenesis, and promote drug resistance (60). Thus, CAFs are therapeutic targets, and research has indicated that the miR-630/KLF6/NF-kB signaling pathway in CAFs may be targeted for treating OC (61). Previous studies have shown that TAMs release anti-inflammatory mediators and angiogenic factors, which suppress anti-tumor immune responses and promote tumor growth (62, 63). However, our findings were not in line with this notion. TAMs are considered M2-like macrophages that exert a tumor-promoting effect. They were recently shown to be in a state of constant transition between M1 and M2 polarization states (64). M1 macrophages participate in the anti-tumor immune response during the early stages of cancer development, whereas M2 macrophages suppress adaptive immunity in advanced tumors, thereby promoting tumorigenesis (64). The proportion of various macrophage phenotypes in the TAM population is regulated by various signaling factors within the tumor microenvironment (65, 66). However, the detailed mechanisms of M1–M2 dynamic transitions remain unclear, necessitating further research into the specific role of TAMs in SOC. Gene set enrichment analysis (GSEA) and KEGG pathway enrichment analysis yielded immune-related molecular mechanisms potentially implicated in SOC. The IL2-STAT5 signaling pathway was previously reported to be involved in the inhibition of T cell proliferation in OC (67). The combined use of an IL6-JAK-STAT3 signaling pathway inhibitor and paclitaxel reduced OC stem cell viability and suppressed tumor growth (68). Further, it has been shown that B-cell receptor signaling plays an important role in the pathogenesis and development of chronic lymphocytic leukemia (69). In-depth exploration of these pathways in SOC will help identify biomarkers and drug targets.

We also investigated the differences in drug sensitivity between the high-risk and low-risk groups, with the results showing that patients in the latter group were more sensitive to cisplatin and oxaliplatin (**Figure 6F**). In contrast, high-risk group patients tended to be more sensitive to tamoxifen. Cisplatin is currently used as a first-line chemotherapy drug for SOC. Unfortunately, with the increase in cisplatin chemotherapy cycles, the risk of platinum resistance or allergies also increases (70). Oxaliplatin is a third-generation platinum derivative that is mainly used alone or along with other platinum drugs for treating SOC relapse. Only partial cross-resistance is observed between it and cisplatin, and thus combination therapy can reduce chemotherapy resistance (71). The estrogen receptor (ER) is upregulated in many patients with OC and is a potential target for endocrine therapy. Tamoxifen is a selective ER modulator that is well-tolerated and has low toxicity (72, 73). Many long-term studies have proven its efficacy for SOC. However, it is still debatable whether tamoxifen can be used as the first-line therapy for treating SOC (73). In this study, we identified potential drugs for treating high-risk patients based on the m^6^A/m^1^A/A-I/APA-LPR model by using the LncMAP database. Among 18 drugs, we found that PD-0325901 and AZD6244 had the most interactions with RNA modification writer-related lncRNAs. Clinical studies have shown the efficacy of both drugs for treating SOC (74, 75). Finally, we established a nomogram involving our model and validated its predictive potential for SOC patient prognosis.

A recent study established a risk model based on four lncRNAs that are involved in m^6^A regulation (AC010894.3, ACAP2-IT1, CACNA1G-AS1, and UBA6-AS1). The model successfully predicted the OS and treatment response in OC patients (45). We checked the expression of those lncRNAs using our data (**Supplementary Figure 6A**) and performed the relationship of those lncRNAs with RNA modification enzymes (**Supplementary Figure 6B**). Similar to a previous study (45), we found that lncRNAs were related to many RNA-modification enzymes. Additionally, we found that ACAP2-IT1 had a significantly positive correlation with RBM15, consistent with previous research (45). Interestingly, we discovered that AC010894.3 was associated with ADARB1 (A-I writer) in addition to m^6^A (METTL5). Moreover, these four lncRNAs were also correlated with different types of RNA modification writers (**Supplementary Figure 6B**), which indicated that lncRNAs may be regulated by multi-RNA modification and the biological functions of lncRNAs may be the result of cross-talk of various RNA modification enzymes. This further suggests that more modification types and related modification enzymes should be included in future studies to determine the relationship between RNA modification and lncRNA regulation more comprehensively. Considering that the interactions of multiple RNA modifications are involved in the incidence and development of SOC (76, 77), we established a risk model based on lncRNAs related to writers of four RNA modification types (m^6^A, m^1^A, APA, and A-I). Therefore, we believe that our model is more reliable and accurate. Nevertheless, the current study has certain limitations. First, the number of SOC samples in TCGA data was not enough, necessitating the use of more datasets to validate the prognostic value of our m^6^A/m^1^A/A-I/APA-LPR model. Second, some of the selected lncRNAs have not yet been explored in the context of cancer, warranting research into their biological function in SOC. Third, several other types of RNA modifications exist, and their effector proteins are not just writers. It is becoming increasingly evident that cross-talk exists among different modification types. Thus, analyzing the incorporation of more RNA modifications, such as m^5^C and m^7^G, will further reveal the regulatory role of different RNA modifications in genes. In a future study, we will further explore the crosstalk of other RNA modification types (such as m^5^C and m^7^G) and other effector (readers and erasers) in SOC. In summary, we established a lncRNA-based risk model that could accurately predict the prognosis of SOC patients and analyzed its association with the tumor microenvironment of SOC. The m^6^A/m^1^A/A-I/APA-LPR model might be a promising prognostic tool for guiding the personalized treatment of SOC.

## Data Availability Statement

The datasets presented in this study can be found in online repositories. The names of the repositories can be found in the article/**Supplementary Material**.

## Author Contributions

GG and XX conceived, designed, and supervised the study. LY performed bioinformatic analyses. LY, KP, SuF, and GG wrote the manuscript. GG and XX revised the manuscript. SW, SC, ST, NW, HZ, XT, XS, ShF, DX, RZ, and YH, collected the data. All authors listed have made a substantial, direct, and intellectual contribution to the work and approved it for publication.

## Funding

This work was supported by grants from the Natural Science Foundation of Zhejiang (Grant No: LQ20C060003), the Health Commission of Zhejiang (Grant No: 2019KY458), and the Wenzhou Public Welfare Science and Technology Project (Grant No: Y20170013).

## Conflict of Interest

The authors declare that the research was conducted in the absence of any commercial or financial relationships that could be construed as a potential conflict of interest.

## Publisher’s Note

All claims expressed in this article are solely those of the authors and do not necessarily represent those of their affiliated organizations, or those of the publisher, the editors and the reviewers. Any product that may be evaluated in this article, or claim that may be made by its manufacturer, is not guaranteed or endorsed by the publisher.

## Supplementary Material

Supplementary Figure 1 Flowchart of this study.

Supplementary Figure 2 Expression of m^6^A/m^1^A/A-I/APA-LPR model-associated lncRNAs in SOC. **(A)** The expression level (fragments per kilobase of exon model per million mapped fragments, FPKM) of six lncRNAs in this study were showed based on our data. **(B)** The expression level of these six lncRNAs were further checked in the TANRIC database (an open-access webapp for interactive exploration of lncRNAs in cancer) based on the normalization data of reads per kilobase per million mapped reads (RPKM). **(C)** qRT-PCR was conducted on RNA samples from six OC cell lines (A2780, A2780-CIS, SKOV3, SKOV3-CIS, CAOV3, and OVCAR3) and one ovarian epithelial cell line (IOSE-80).

Supplementary Figure 3 Kaplan–Meier survival analysis. Survival analysis of SOC patients grouped based on the expression of RNA modification writer-related lncRNAs in the training set.

Supplementary Figure 4 Difference in hallmark gene signatures, KEGG pathway enrichment, and the tumor immune microenvironment between low- and high-risk patient groups in the entire set. **(A)** GSEA. **(B)** KEGG pathway enrichment analysis.

Supplementary Figure 5 Assessment of the writer-related lncRNA model and clinical features in the entire set. **(A)** Univariate and multivariate Cox regression analyses of the clinical characteristics and risk model with regard to OS. **(B)** ROC curves of the model and clinical characteristics. **(C)** Time-dependent ROC curves to evaluate the predictive accuracy of the m^6^A/m^1^A/A-I/APA-LPR score and other clinicopathological parameters for 1-, 2- and 3-year OS of SOC patients in the entire set. (D) Concordance indexes of model and clinical characteristics.

Supplementary Figure 6 The correlation between four lncRNAs from a previous study and RNA modification writers of m^6^A, m^1^A, APA, and A-I. **(A)** The expression level (FPKM) of four lncRNAs based on our data. **(B)** Correlation analysis of these four lncRNAs with writers of m^6^A, m^1^A, APA, and A-I.
