# Supplementary figures and images for: Four Types of RNA Modification Writer-Related lncRNAs Are Effective Predictors of Prognosis and Immunotherapy Response in Serous Ovarian Carcinoma
Source: Front Immunol. 2022 May 2;13:863484. doi: 10.3389/fimmu.2022.863484 (PMC9108167; doi:10.3389/fimmu.2022.863484)

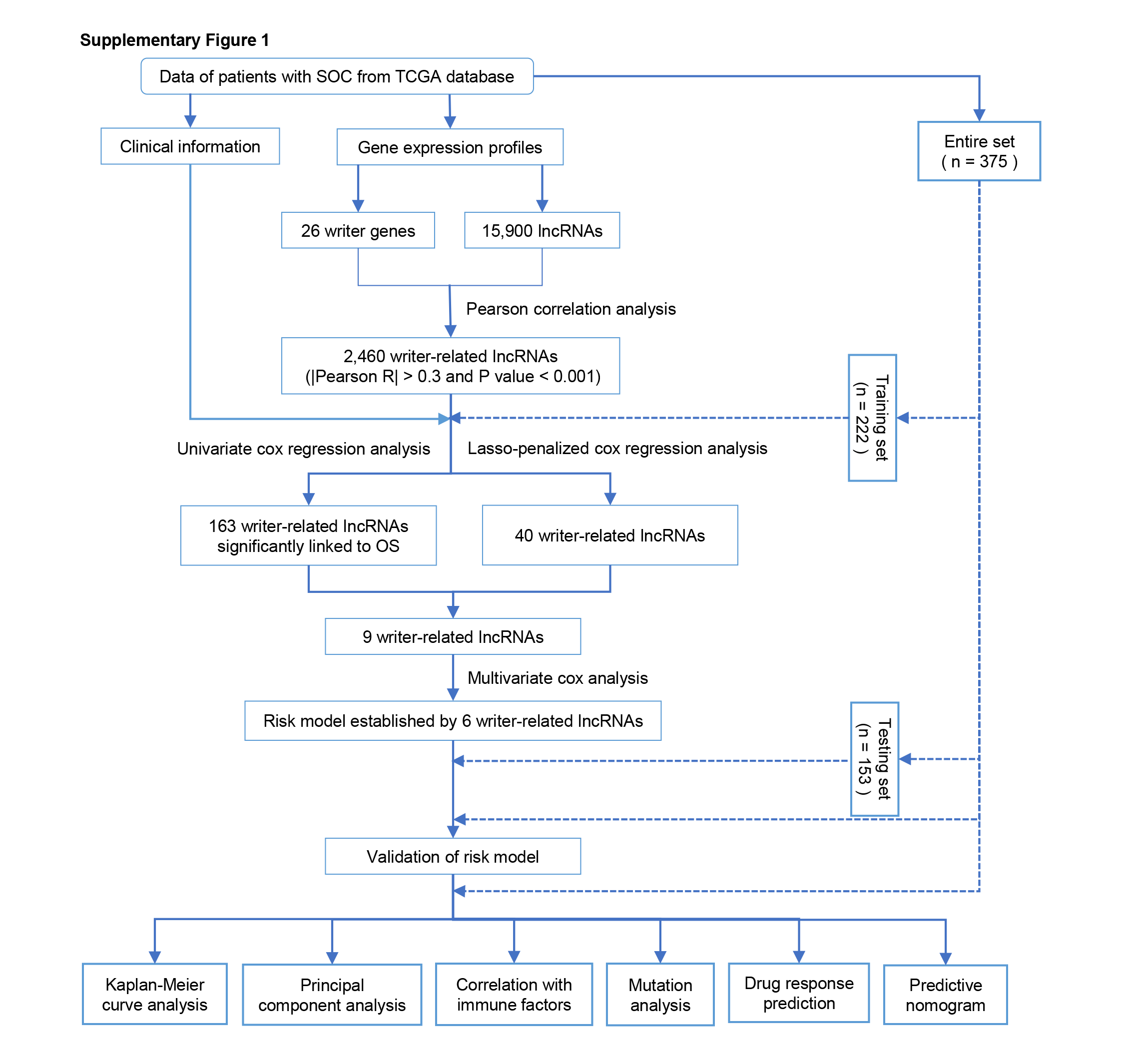

Supplement: Supplementary Figure 1 — Flowchart of this study. [file Image_1.tif]

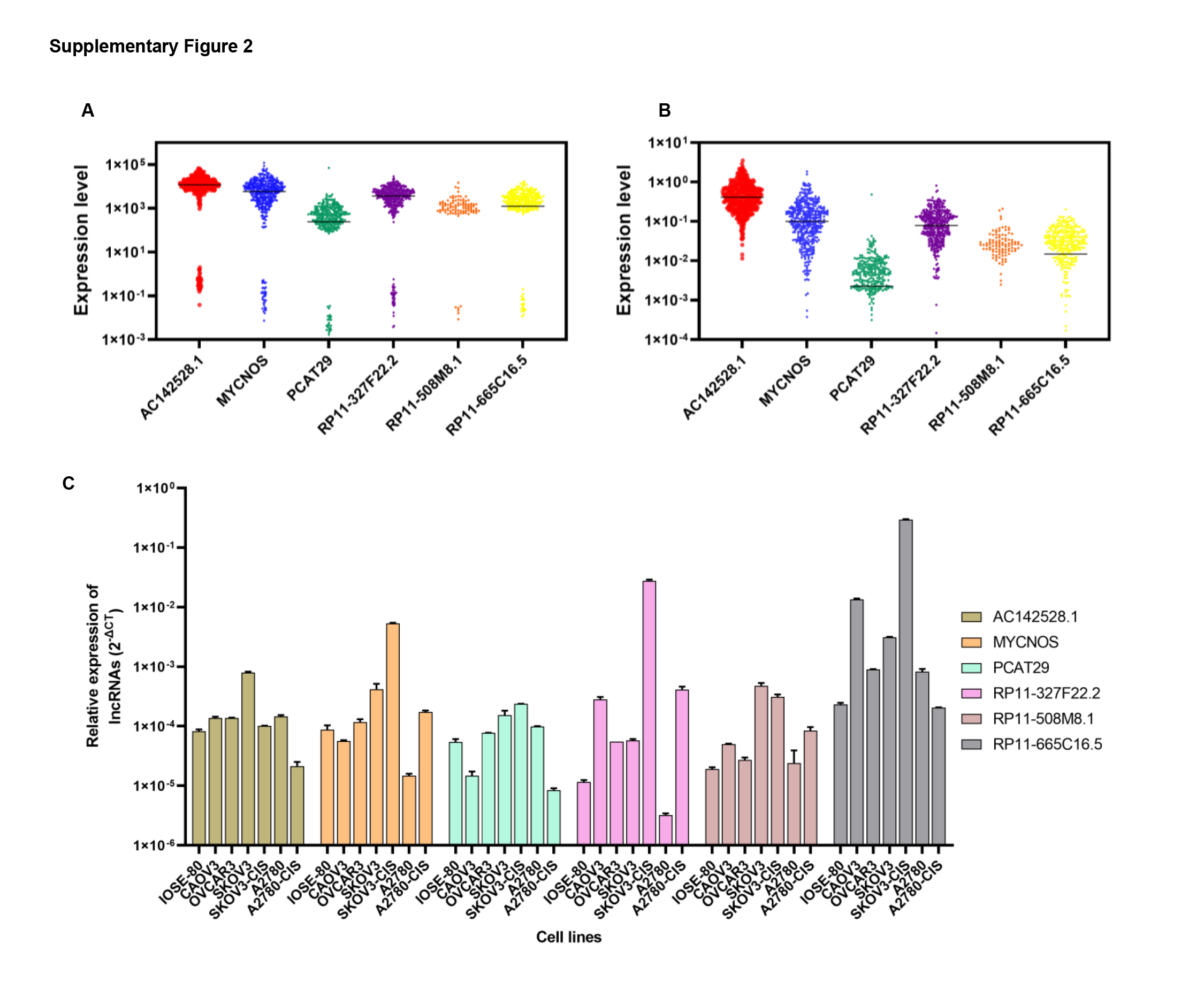

Supplement: Supplementary Figure 2 — Expression of m6A/m1A/A-I/APA-LPR model-associated lncRNAs in SOC. (A) The expression level (fragments per kilobase of exon model per million mapped fragments, FPKM) of six lncRNAs in this study were showed based on our data. (B) The expression level of these six lncRNAs were further checked in the TANRIC database (an open-access webapp for interactive exploration of lncRNAs in cancer) based on the normalization data of reads per kilobase per million mapped reads (RPKM). (C) qRT-PCR was conducted on RNA samples from six OC cell lines (A2780, A2780-CIS, SKOV3, SKOV3-CIS, CAOV3, and OVCAR3) and one ovarian epithelial cell line (IOSE-80). [file Image_2.tif]

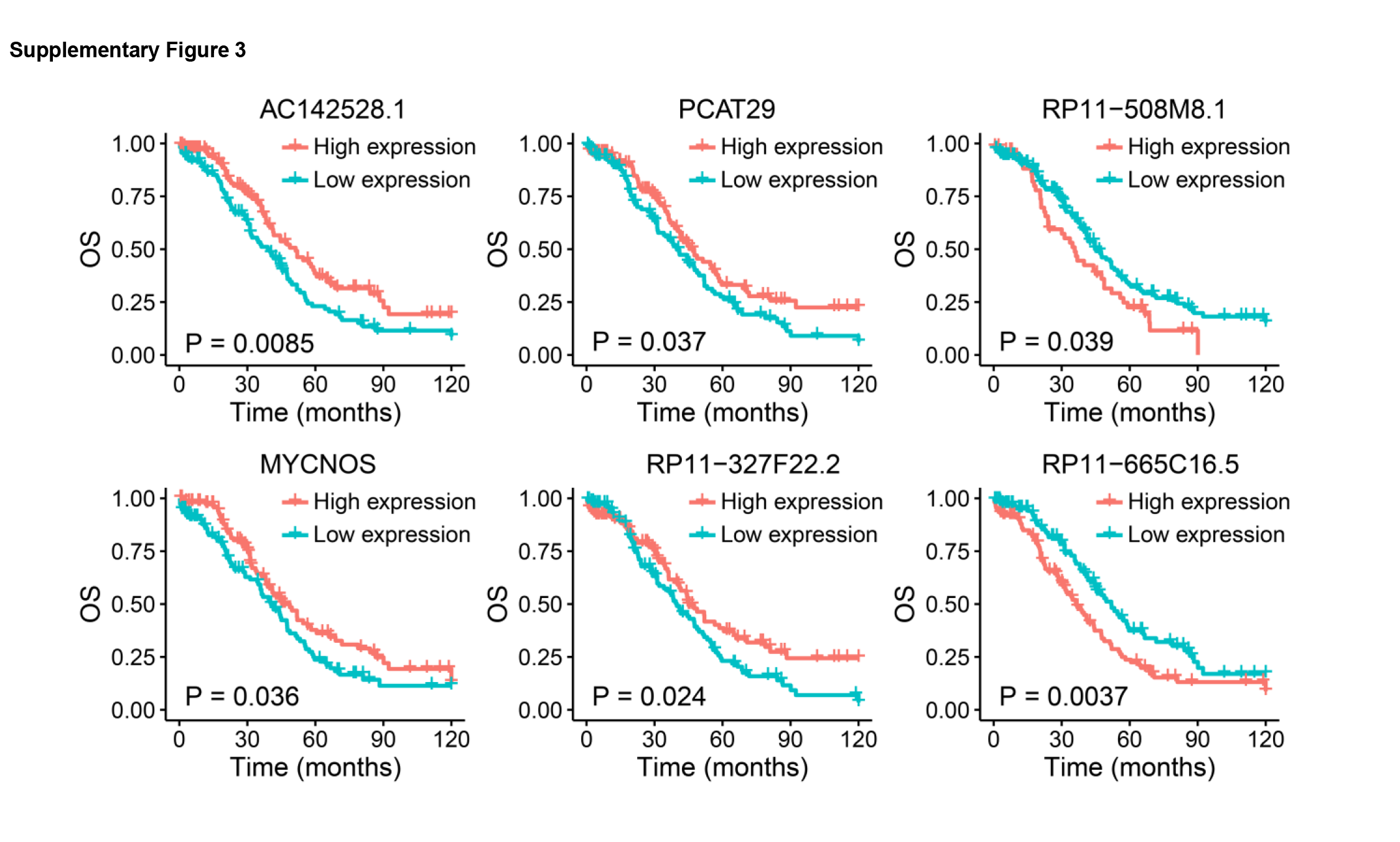

Supplement: Supplementary Figure 3 — Kaplan–Meier survival analysis. Survival analysis of SOC patients grouped based on the expression of RNA modification writer-related lncRNAs in the training set. [file Image_3.tif]

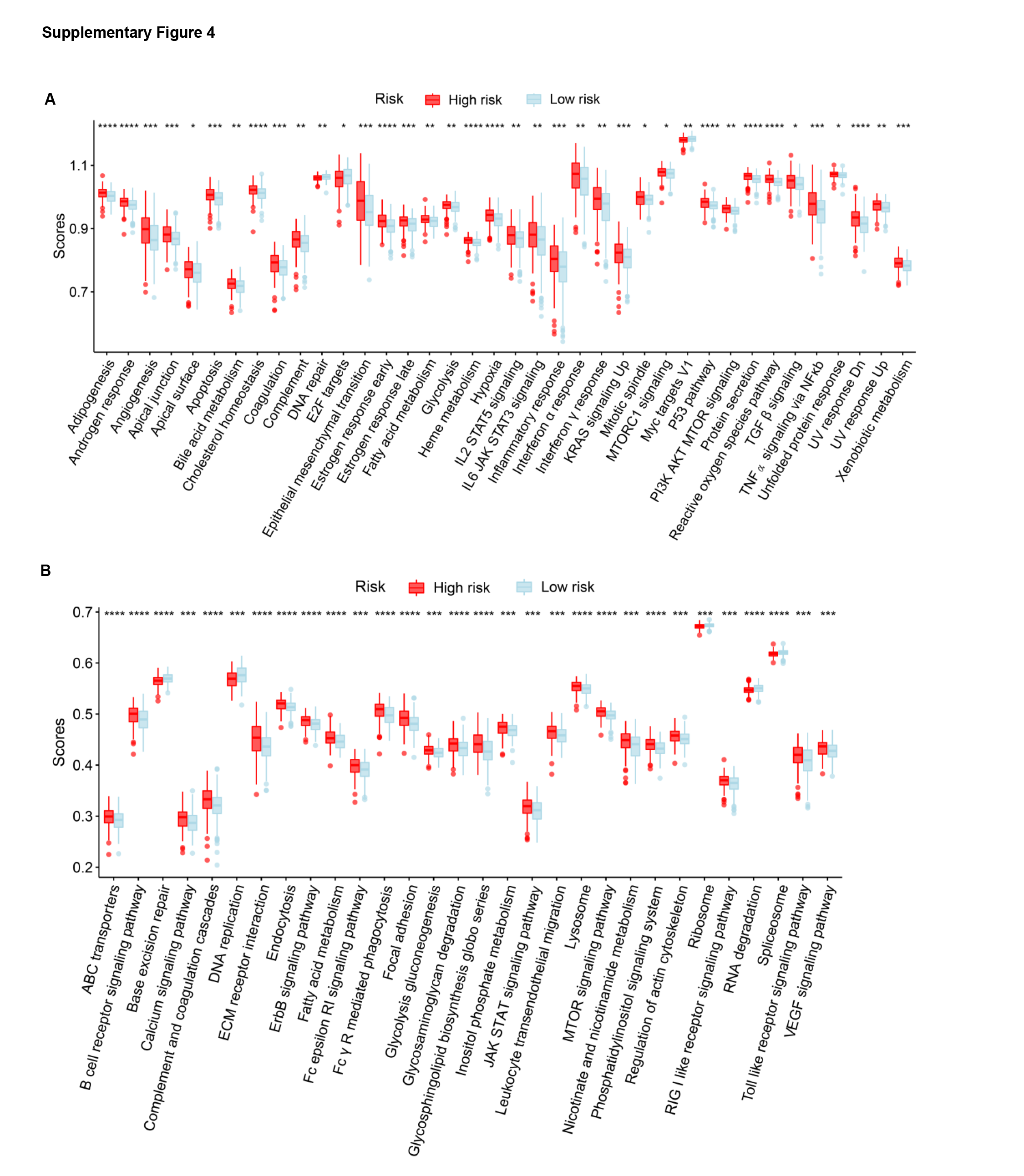

Supplement: Supplementary Figure 4 — Difference in hallmark gene signatures, KEGG pathway enrichment, and the tumor immune microenvironment between low- and high-risk patient groups in the entire set. (A) GSEA. (B) KEGG pathway enrichment analysis. [file Image_4.tif]

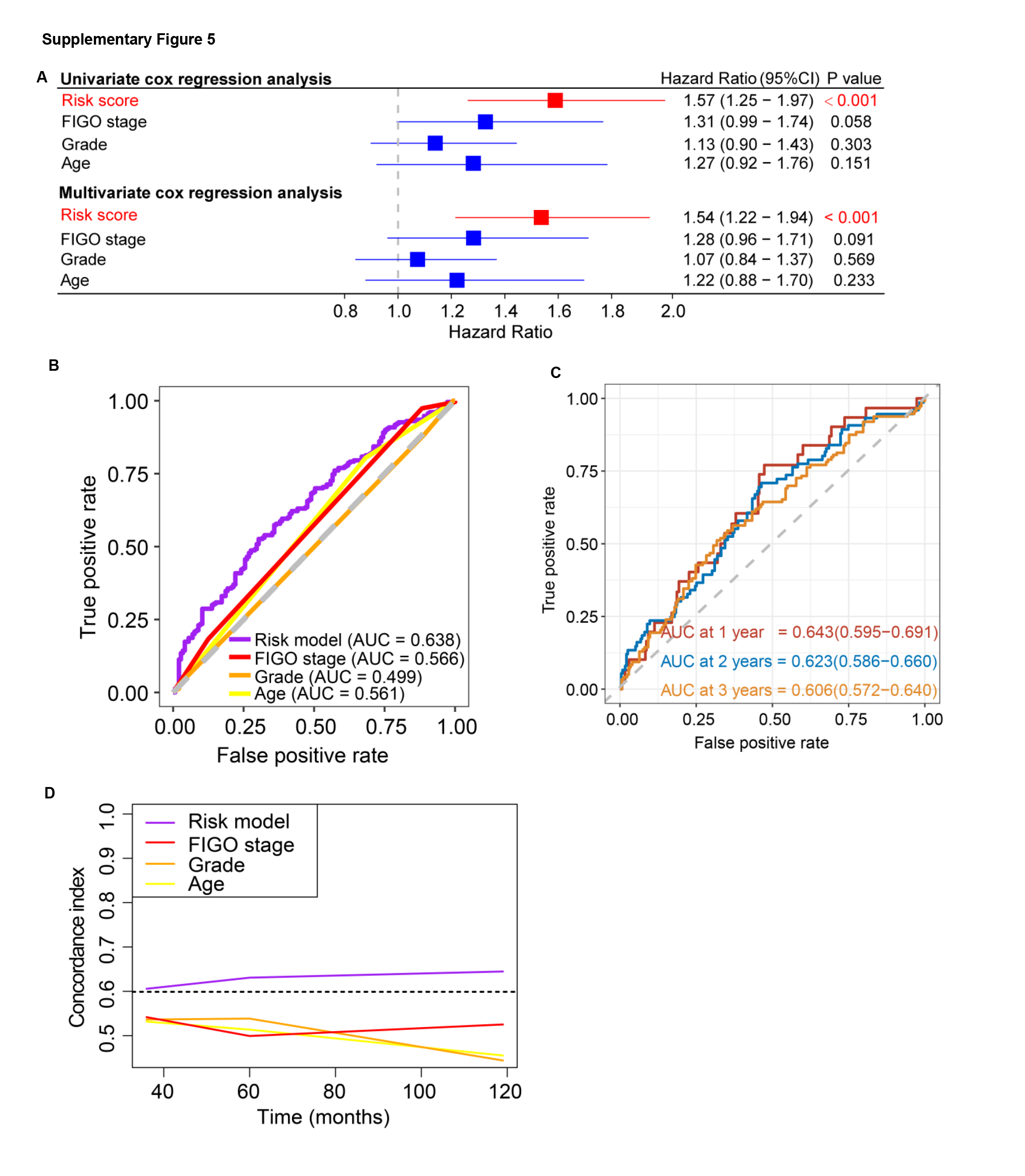

Supplement: Supplementary Figure 5 — Assessment of the writer-related lncRNA model and clinical features in the entire set. (A) Univariate and multivariate Cox regression analyses of the clinical characteristics and risk model with regard to OS. (B) ROC curves of the model and clinical characteristics. (C) Time-dependent ROC curves to evaluate the predictive accuracy of the m6A/m1A/A-I/APA-LPR score and other clinicopathological parameters for 1-, 2- and 3-year OS of SOC patients in the entire set. (D) Concordance indexes of model and clinical characteristics. [file Image_5.tif]

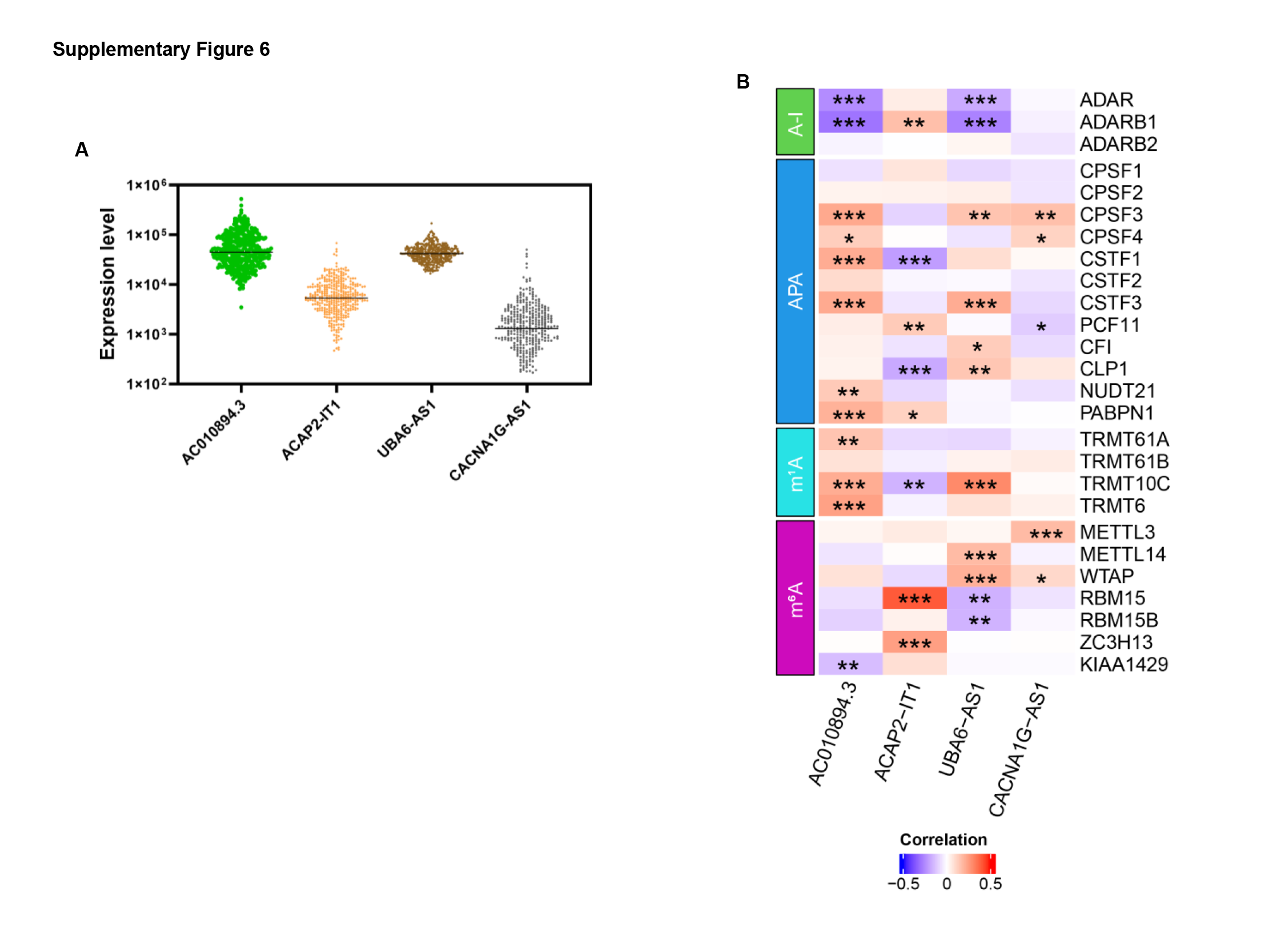

Supplement: Supplementary Figure 6 — The correlation between four lncRNAs from a previous study and RNA modification writers of m6A, m1A, APA, and A-I. (A) The expression level (FPKM) of four lncRNAs based on our data. (B) Correlation analysis of these four lncRNAs with writers of m6A, m1A, APA, and A-I. [file Image_6.tif]
